# Impact of different levels of handling on *Solea senegalensis* culture: effects on growth and molecular markers of stress

**DOI:** 10.1007/s10695-023-01239-9

**Published:** 2023-09-21

**Authors:** David G. Valcarce, Marta F. Riesco, Juan Manuel Martínez-Vázquez, José Luis Rodríguez Villanueva, Vanesa Robles

**Affiliations:** 1 Cell Biology Area, Molecular Biology Department, Universidad de León, Campus de Vegazana s/n, 24071 León, Spain; 2 Instituto Español de Oceanografía, Centro Oceanográfico de Santander (COST-IEO), CSIC, Calle Severiano Ballesteros 16, 39004 Santander, Spain; 3 Instituto Galego de Formación en Acuicultura, Xunta de Galicia, Illa de Arousa, 36626 Pontevedra, Spain

**Keywords:** *Solea senegalensis*, Stress, Growth, Cortisol, Handling, Glucocorticoid receptors

## Abstract

Aquaculture routine practices may cause stress induction on the fish and compromise their welfare affecting the production. This experiment aimed to evaluate the potential links between handling during culture with stress responses and growth on Senegalese sole (*Solea senegalensis*). We worked with two fish cohorts in terms of initial body weight and culture stage: Trial 1 included specimens in the fattening stage (226 ± 4.96 g) and Trial 2 animals in the pre-fattening stage (27.20 ± 0.44 g). The tested culture protocol, which lasted 6 and 4 months for Trial 1 and 2, respectively, mainly reduced handling-derived stressors in the experimental tanks via lowering routine samplings to a minimum. This decrease of the handling-derived stress was reflected in both trials with lower concentration of circulating cortisol in blood plasma from the experimental fish when compared to controls. Moreover, the proposed protocol promoted higher growth in the fish cultured in the less disturbing protocol in Trial 2. Higher specific growth rates and mean body weight and length were reported. In order to further explore the potential beneficial effects of our protocol, we studied the musculoskeletal from Trial 2 gene expression of key genes regulating glucocorticoid signaling pathway and apoptosis: glucocorticoid receptors 1 and 2 (*gr1*, *gr2*), heat shock protein 90 AA (*hsp90aa*), and caspase 6 (*casp6*). In line with the cortisol reduced level in this trial, *gr1*, *hsp90aa*, and *casp6* genes showed lower expression in the samples coming from the experimental group. The findings of this study provide valuable information to the aquaculture industry for the management of *Solea senegalensis* stress and welfare.

**Supplementary Information:**

The online version contains supplementary material available at 10.1007/s10695-023-01239-9.

## Introduction

Aquaculture husbandry practices and derived welfare issues are becoming exponentially focused on by scientific community, consumers, and policy makers. Research approaches to evaluate animal welfare are continually developing and are crucial towards an optimized aquaculture production. The actual challenges for the international scientific community include the generation of optimized culture protocols for a vast diversity of cultured fish species under international and national legislative frameworks. As an example within the strategic guidelines for a more sustainable and competitive EU aquaculture for the period 2021 to 2030 (European Commission 2021) it is pointed out the need of “further research and innovation, in particular on species-specific welfare parameters, including nutritional needs in different rearing systems.” Moreover, fish welfare is a key issue for the industry, not only for consumer’s perception or acceptance but also in terms of productivity (Ashley 2007). Within the different approaches towards improving fish welfare, the avoidance of the maladaptive consequences of chronic stress has been a central welfare aim during last decades (Barton and Iwama 1991). In their natural environment, fish have to bear many stressors: presence of predators, seasonal food shortages, biochemical water fluctuations, or social conflicts. In aquaculture inland facilities, many of these sources of stress change. Food availability is constant and the physicochemical conditions of the water are stable and monitored. However, as a result of industrial activity, the continuous presence of humans and handling at multiple instances during routine procedures (feeding, tank cleaning, population assessment, periodic control sampling, etc.) becomes as a stress source.

Teleost fish, as other vertebrates, have evolved both constitutive and inducible mechanisms for deal with stressors. The primary stress response in teleost fish involves the activation of the hypothalamic–pituitary–interenal (HPI) axis driven by the release of catecholamines. Hypothalamic corticotropin releasing factor stimulates pituitary corticotropic hormone synthesis, which once released, in turn promotes cortisol synthesis and mobilization from by steroidogenic cells located in the interrenal glands (Faught and Vijayan 2016). Cortisol together to catecholamines inductee secondary and tertiary stress responses which finally alter fish homeostasis in many ways. The effects derived from stress are very diverse and can ultimately affect key subjects in aquaculture production such as the health, growth, or reproduction (Schreck and Tort 2016).

In this experiment we work with the species *Solea senegalensis*. The quality characteristics of the flesh the *Soleidae* family makes it economically remarkable mainly in southern Europe with a total production in the UE of 8.715 Tons during 2014–2020 according to the European Aquaculture Production Report 2014–2020 (FEAP, 2021). During the last decades, the scientific community has strived to develop optimized culture protocols for this species and also common sole (*Solea solea*), both highly valued by the consumer. Multiple research groups have focused on different research lines: from the development of diets with optimized nutritional content (Silva et al. 2010; Canada et al. 2019) in each vital stage, to reproduction management (Rasines et al. 2012; Riesco et al. 2017) or to zootechniques improvements (Martín et al. 2017; Ibarra-Zatarain et al. 2020). The study of the effect of different kind of stress and potential methods to reduce them in this species has also been addressed in a variety of experimental designs addressing the impact of thermal stress (Benítez-Dorta et al. 2017), osmotic challenge (Arjona et al. 2007), or pathogen resistance (Peixoto et al. 2018) among others. To explore whether the welfare status of the fish can be modulated by optimizing the culture conditions and if this modulation could have benefits in terms of production arise as an important question of undoubtedly interest for the aquaculture industry. Thus, we hypothesized that under the same feeding regime, a lower human-handling culture protocol might positively affect fish welfare and growth by reducing anthropogenic sources of stress in aquaculture facilities. The goal of this study was, therefore, to evaluate the potential links between different levels of handling-derived stressors with molecular stress responses (measurement of plasma cortisol levels and gene expression of stress biomarkers) and animal growth in two Senegalese sole cohorts in terms of life stages, in order to provide data on fish welfare assessment and its direct impact on their culture.

## Material and methods

### Ethics approval

All experimental procedures on fish were authorized by the Bioethics and Animal Welfare Committees of the facilities under the project registration number ULE009-2020 and were conducted following the Spanish and European regulations for the use of laboratory animals.

### Experimental design and Senegalese sole rearing conditions

In order to validate our hypothesis, we carried out two trials with different cohorts of specimens: Senegalese soles in the fattening phase (Trial 1) and in the pre-fattening stage (Trial 2). In both sub-experiments, two experimental groups were generated: the control group (CTRL), that was subjected to routine husbandry according to their production stage and the experimental group (EXP), that was cultured under experimental husbandry conditions with a reduced handling protocol. Fish were maintained in RAS (Trial 1) and open flow circuit system (Trial 2) in tanks adequate to their size (Table 1), with a water renovation and constant moderate aeration. An artificial photoperiod of 14 h light and 10 h dark was used throughout the entire experiment. Temperature varied according to external conditions (Table 1). Generally, a monthly biometric evaluation is carried out in fish aquaculture facilities to adjust the feed dose to the changing biomass of the tank therefore control groups were sampled every month. Fish in EXP groups were exposed to lower amounts of tactile, visual, olfactory, and auditory stimuli derived from human interaction during handling protocols. The decrease of these anthropogenic-derived stimuli was mainly based by the reduction of handling and human occurrence near the tanks. Biometric evaluations involve fish withdraws from the tanks and the measurements include all of these stimuli directly from the personnel or the material used. The behavior of the animals and their appearance were monitored daily in all the tanks involved in the experiment during feeding periods (twice per day), with special interest in the specimens subjected to the experimental conditions of Trial 2 to assure animal welfare. Logically, under the experimental conditions in Trial 2 more detritus were accumulated at the bottom of the tank as the days passed between cleanings. However, the used tanks allowed maintaining acceptable hygienic culture conditions in the experimental individuals. Details on experimental procedures adapted to the specific production stage are described in each trial:

**Table 1:** Culture conditions in the experiment

Trial	Culture stage	Fiberglass tank type volume (m^3^ and surface (m^2^))	Initial biomass (g)	Photoperiod (h; light:dark)	T (°C)	Food dose (% biomass)
1	Fattening	0.5 m^3^ square (1 m^2^)	816.1 ± 10.25	14:10	19 ± 1	0.5
2	Pre-fattening	0.2 m^3^ circular (0.70 m^2^)	33.90 ± 0.48	14:10	17.27 ± 0.22	1.5

**Fig. 1:**
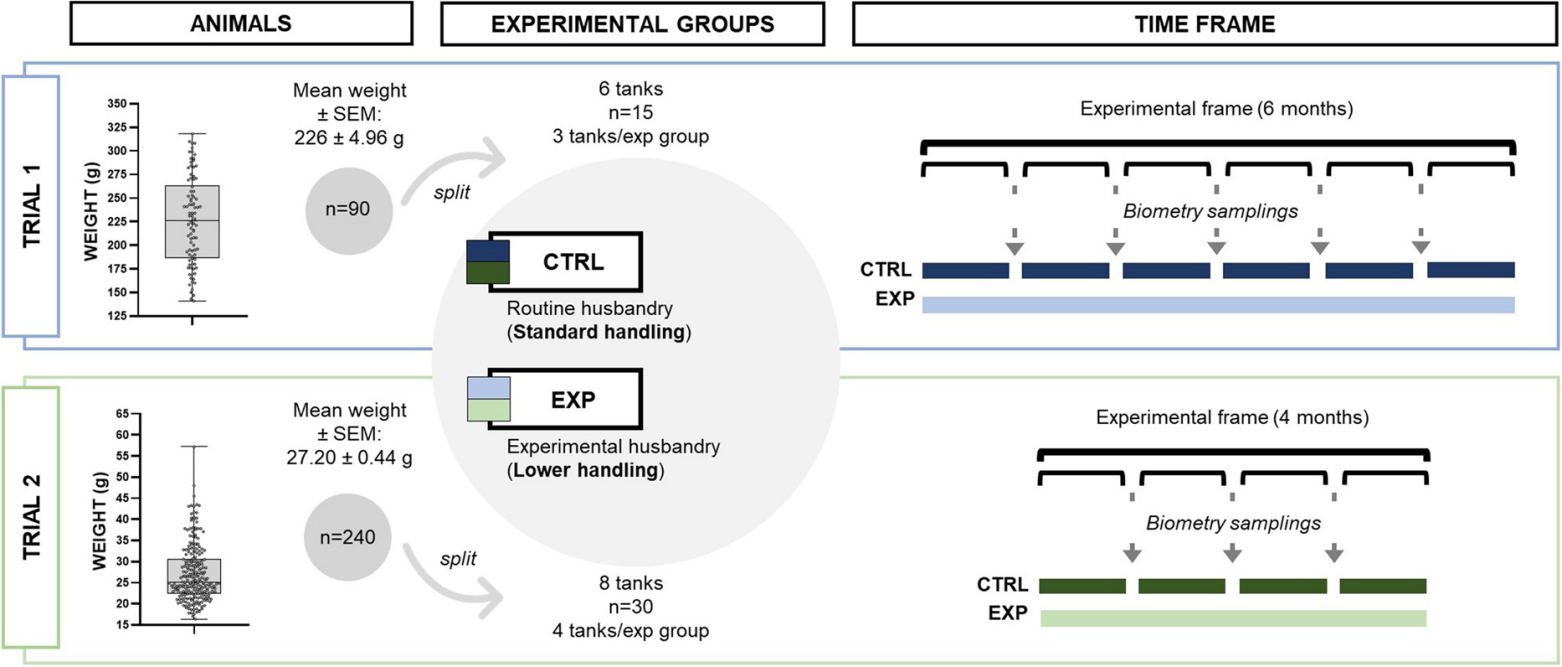
Experimental design for the evaluation of the impact of a lower human-animal culture protocol in *Solea senegalensis*. Two trials were performed: Trial 1 corresponds to specimens in the fattening culture stage and Trial 2 corresponds to specimens in the pre-fattening stage. CTRL refers to the animal standard cultured. EXP refers to the animals cultured under a lower handling protocol. General information about initial populations, tank replicates and *n* as well as sampling planning are included

Trial 1 took place during 6 months in the facilities of the *Instituto Galego de Formación en Acuicultura* (Illa de Arousa, Spain). Senegalese soles in the fattening phase (initial pooling weight of 226 ± 4.96 g) were used (Fig. 1). Animals were fed with commercially prepared fish food: LE-5 Europa RG (Skretting S.A., Spain). Each experimental group consisted in 3 tank replicates (*n* = 15 fish). CTRL groups were subjected to a total of 6 withdraws from the tanks including biometric measurements. In the EXP group only 2 biometric samplings (initial and final) were performed. Culture conditions are in Table 1.

Trial 2 was carried out during 4 months at the facilities of the Marine Culture Plant *El Bocal* of Spanish Institute of Oceanography-CSIC (Santander, Spain). The involved specimens were in the pre-fattening stage (initial pooling weight of 27.20 ± 0.44 g; Fig. 1). Animals were fed with commercially prepared fish food: LE-2 and LE-3 Europa RG (Skretting S.A., Spain) pellets. Each group included 4 tank replicates (*n* = 30). Culture conditions are included in Table 1.

**Fig. 2:**
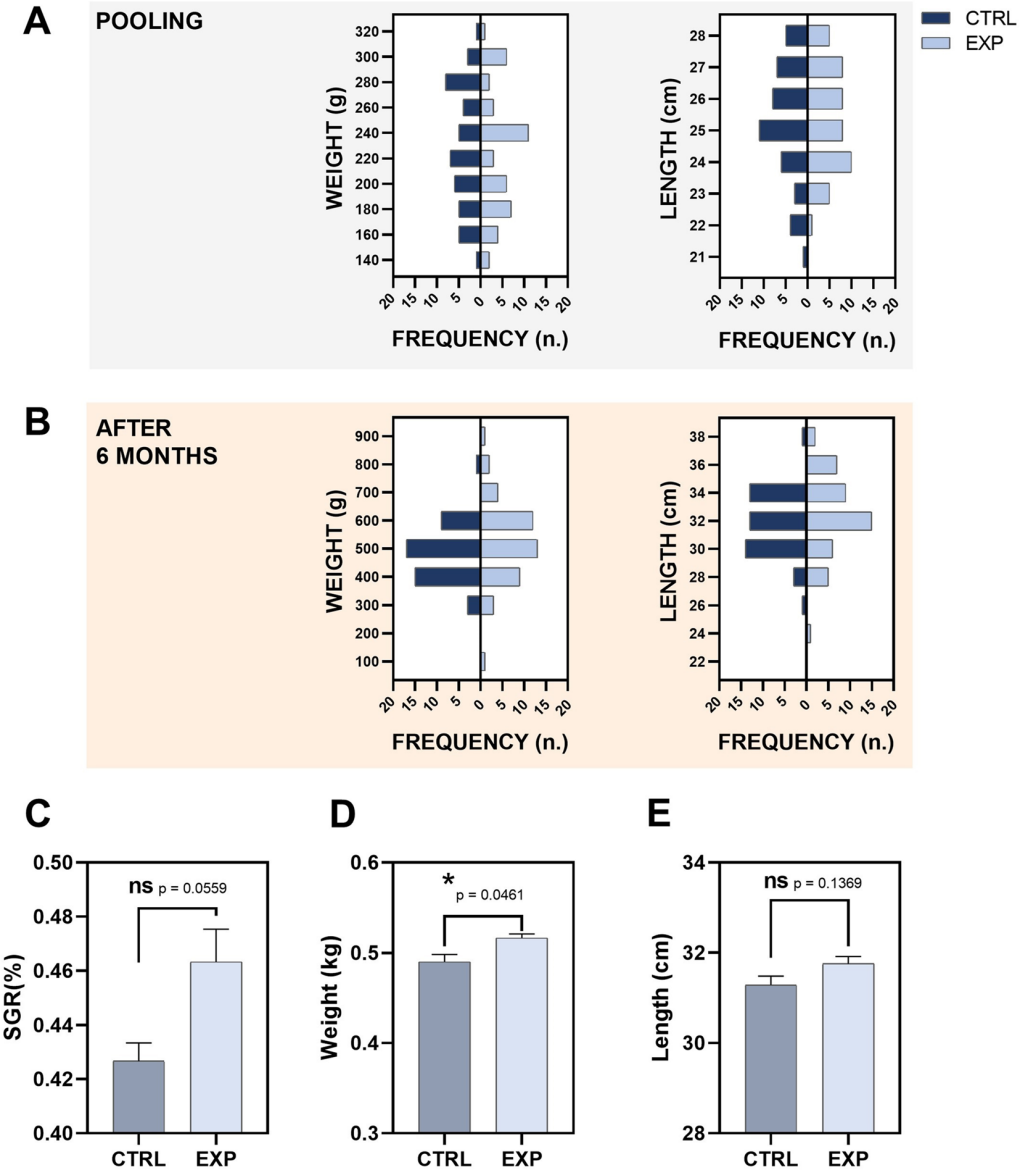
Biometry and growth data from Trial 1 corresponding to the animals in the fattening culture stage. Weight and length histograms at pooling (*t* = 0 months; **A**) and at the end of the experiment (*t* = 6 months; **B**) are included (*n* = 45; 15 fish/tank). Specific growth rate (%) values in each experimental group are plotted in **C** (*n* = 3 tanks). Mean body weight (**D**) and length (**E**) of the tanks included in each experimental group are represented. CTRL refers to the animal standard cultured. EXP refers to the animals cultured under a lower handling protocol. ns denotes no statistically significant difference. Asterisks show statistically significant difference between groups

CTRL tanks were subjected to 4 iterations and in EXP groups group, only 2 biometric samplings (initial and final) were performed (Fig. 2). Tank cleaning was also reduced to once per week in the EXP group, while in the CTRL group the tanks were siphoned daily.

### Biometry and animal growth

To evaluate the effects of the two experimental conditions on fish growth, in the samplings (monthly in the CTRL group, twice in the EXP group), all fish within the tanks were weighted and measured following routine protocols and manipulation. Growth was reported as specific growth rate (*SGR*, percent change in body weight per day) and was calculated as follows:

$$\textrm{S} GR\left(\%\right)=100\times \frac{lnWf- lnWi}{t}$$

where *Wf* and *Wi* are, respectively, the final and initial mean weights (g) in each tank and *t* is period (days).

We also evaluated feed conversion ratio (*FCR*) in order to compare energy use under different handling conditions. FCR was calculated as:

$$FCR=\frac{Feed}{Bf- Bi}$$

where *Feed* corresponds to the total amount of feed (g) provided to the tanks during the experiment and *Bf* and *Bi* are, respectively, the final and initial biomass (g) in each tank.

### Blood samplings and plasma cortisol levels analysis

For blood recovery in the final samplings, two methods were used varying on the trial due to fish age/size and therefore resistance to manipulation in order to avoid cortisol increase due to handling during sampling. In Trial 1, animals (*n* = 7/group) were individually withdrawn from the tank and immediately killed by decapitation at any case before 2 min of tank extraction. Blood samples were recovered from the exposed branchial area using a 1 mL heparinized syringe. In Trial 2, also within the first 2 min after the tank withdrawn, animals (*n* = 8/group) were immobilized using a wet baize and blood was extracted from the dorsal artery with a 0.3 mL heparinized syringe. Immediately after, fish were terminally anaesthetized with tricaine overdose. In both sub-experiments, blood was transferred to 1.5 mL microcentrifuge tubes containing 10 μL of heparin. Afterwards, samples were centrifuged (10000 × g for 3 min at 4 °C) and plasma fraction was recovered and stored at −80 °C until assayed.

Plasma cortisol concentration was measured using cortisol enzyme immunoassay kit #500360 (Cayman Chemical, Ann Arbor, MI). Following the manufacturer’s recommendations, each sample was run in duplicate and cortisol concentrations were calculated relative to the intraassay standard curve.

### RNA extraction and reverse transcription

RNA extraction from muscle samples was performed using TRIzol Reagent (Invitrogen) according to the manufacturer’s instructions. Tissue disruption and cell lysis was facilitated by mechanic disruption. RNA quantity and quality (A260/A280 ratio ranging from 1.8 to 2.0) were checked using a NanoDrop™ One/OneC spectrophotometer (ThermoScientific™). RNA integrity was evaluated on 1% agarose in TAE buffer gel using GelRed^®^ to stain 28S and 18S ribosomal RNA (rRNA) fragments (data not shown). Reverse transcription (1 μg of RNA) was performed using the High-Capacity cDNA Reverse Transcription Kit (Applied Biosystems) following the kit protocol. The resulting cDNA samples were stored at −20 °C prior to qPCR analysis.

### Quantitative real time PCR

The differential expression of muscle target transcripts: glucocorticoid receptor 1 (*gr1*), glucocorticoid receptor 2 (*gr2*), cytosolic heat shock protein 90 AA (*hsp90aa*), and caspase 6 (*casp6*) was measured in muscle samples from 8 soles per group from Trial 2. Target transcripts were selected according to their demonstrated relation to stress response and apoptosis in vertebrates (Roberts et al. 2010; Benítez-Dorta et al. 2017; Spead et al. 2018). Primers used were specific for Senegalese sole and already published. Primer sequences and references are presented in Table 2. mRNAs were analysed by real-time quantitative PCR (qPCR) with a StepOnePlus (Applied Biosystems) thermocycler. Data were normalized using eukaryotic elongation factor 1 alpha 1 (*eef1a1*) as a house-keeping transcript. All replicates were run in triplicate on a 20-μL reaction containing 25 ng of cDNA. Each reaction included 10-μL SYBR green master mix (Applied Biosystems), forward and reverse primers (10 μM) and bidistilled water up to 20 μL. Thermal cycle consisted on an initial activation step of 10 min at 95 °C followed by 40 cycles of 10 s at 95 °C and 60 s at annealing temperature (60 °C). Melting curve analysis was also carried out to determine the specificity of qPCR reactions—one cycle at 95 °C for 15 s, 60 °C for 1 min, slow ramping of temperature to 95 °C, and 95 °C for 15 s. Standard curves for target and reference genes were generated in order to analyse the linearity, detection range, and qPCR amplification efficiency (Table 2) of primer pairs in our samples. Gene expression was calculated following Pfaffl’s mathematical model (Pfaffl 2001).

**Table 2:** Primers used in gene expression analyses by real-time qPCR

Gene	Oligo	Sequence (5′ to 3′)	Tm (°C)	Efficiency (%)	Reference
*eef1a1*	F	GATTGACCGTCGTTCTGGCAAGAAGC	60	94.09	(Infante et al. 2008)
	R	GGCAAAGCGACCAAGGGGAGCAT			
*gr1*	F	CCTGCCGCTTCCACAAGTGTCTGATG	60	101.89	(Benítez-Dorta et al. 2017)
	R	TTCAACTGGTGGAGGTGGCGGTGT			
*gr2*	F	TCAGCGTGGAGTTCCCGGAGATG	60	94.24	
	R	GGTGGAACAGCAGCGGCTTGATG			
*hsp90aa*	F	GACCAAGCCTATCTGGACCCGCAAC	60	97.48	(Manchado et al. 2008)
	R	TTGACAGCCAGGTGGTCCTCCCAGT			
*casp6*	F	CAGGACATCACAGCCATGTT	60	102.37	(Sarasquete et al. 2018)
	R	TCACTGTCCACAGCATCACA			

### Statistical analysis

All data were tested for normality (Shapiro–Wilk test) using GraphPad Prism 9.0.0 package (GraphPad Software, Inc.). Statistically significant differences (*p < 0.0500; **p < 0.0100) between groups were tested using an unpaired Student’s *t* test (variables: SGR (Trial 1 and 2) and *hsp90aa* normalized gene expression); an unpaired Student’s test with Welch’s correction if the variances of the variables were not equal after running a *F* test (variables: cortisol levels (Trial 1 and 2); *casp6* and *gr1* normalized gene expression ) or Mann–Whitney test (variables: mean weight and mean length (Trial 1 and 2) and *gr2* normalized gene expression) in each case for experimental group comparison. All data are shown as mean ± SEM.

## Results

### Biometry and growth performance

Data on biometric data and growth performance from Trial 1 (fattening stage) are reported in Fig. 2. The initial mean body weights and lengths of fish were similar in both groups at the beginning of the experiment, respectively (Fig. 2A). The experimental protocol did not report a clear promotion of fish weight nor length when compared to the control one in the fattening stage. Both groups showed similar population distributions after 6 months of culture (Fig. 2B). As a reflection of this, specific growth rate values pointed to a non-statically significant (*p* = 0.0559) trend increase in EXP group with a mean SGR value of 0.4633 ± 0.0120% in the EXP vs 0.4267 ± 0.0067% in the CTRL group (Fig. 2C). Fish increased body weight around 275 g in 6 months (Fig. 2D; Supplementary material 1) and their length gained around 6 cm (Fig. 2E; Supplementary material 1) finding statistically significant differences in mean body weight in the tanks (Fig. 2D). FCR values for this trial are included in Supplementary material 2. FCR mean values were lower in the EXP group (*p* = 0.0321).

**Fig. 3:**
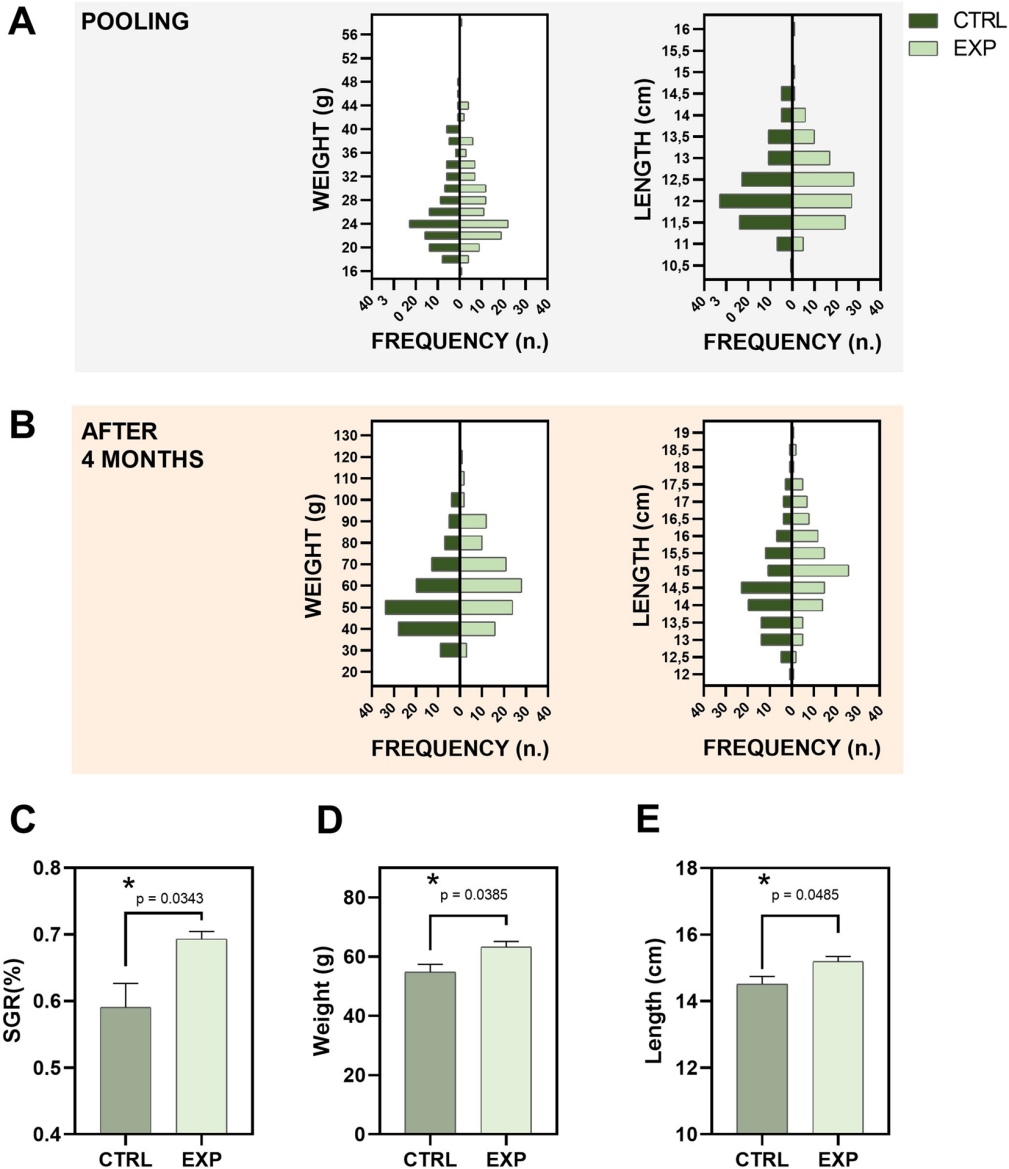
Biometry and growth data from Trial 2 corresponding to the animals in the pre-fattening culture stage. Weight and length histograms at pooling (*t* = 0 months; **A**) and at the end of the experiment (*t* = 4 months; **B**) are included (*n* = 240; 30 fish/tank). Specific growth rate (%) values in each experimental group are plotted in **C** (*n* = 4 tanks). Mean body weight (**D**) and length (**E**) of the tanks included in each experimental group are represented. CTRL refers to the animal standard cultured. EXP refers to the animals cultured under a lower handling protocol. Asterisks show statistically significant difference between groups

Contrary to Trial 1, at the end of the Trial 2 (pre-fattening), mean body weight increased 2.3 times in EXP group vs 2.04 times in CTRL group. Fish grew from around 27 g at pooling (Fig. 3A and D; Supplementary material 1) to 63.30 ± 1.655 g mean final body weight in the EXP group and 54.77 ± 1.559 g in the CTRL group. The histograms included in Fig. 3B clearly describe different populations at the end of the 4 month-trial in each group. The comparison of these mean body weight values in the tanks reported statistically significant differences (*p* = 0.0385). The SGR (%) data from Trial 2 is included in Fig. 3C. While CTRL tank replicates reported a mean growth rate of 0.5906 ± 0.0358%, EXP tanks scored 0.6932 ± 0.0114%. Statistical analysis confirmed (*p* = 0.0343) that the experimental protocol promoted growth when compared to the standard one. Fish length was correspondingly equally modulated by the lower handling protocol in the pre-fattening Trial 2. From the initial mean values around 12.4 cm in both groups (Fig. 3A and E; Supplementary material 1), the length increase was revealed higher (*p* = 0.0485) in the EXP group after 4 months (15.19 ± 0.1197 cm) compared to the CTRL group (14.52 ± 0.1189 cm). In line with SGR and biometrical data, FCR data were statistically significant different in EXP group (*p* = 0.0006; Supplementary material 2).

### Circulating plasma cortisol concentration

**Fig. 4:**
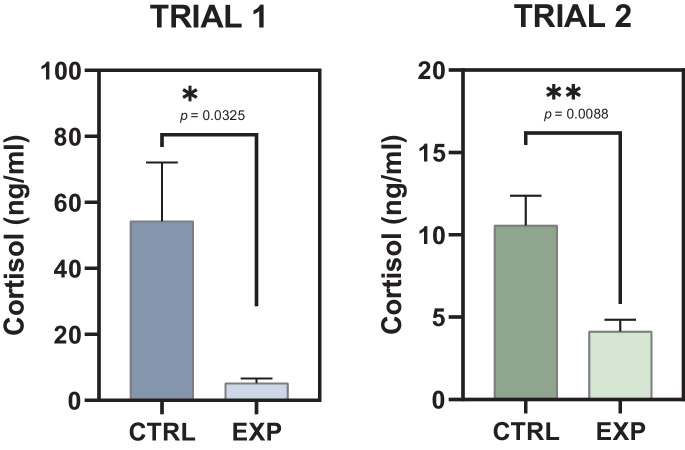
Circulating plasma cortisol concentration for each Trial in the experiment: Trial 1 corresponds to specimens in the fattening culture stage and Trial 2 corresponds to specimens in the pre-fattening stage. CTRL refers to the animal standard cultured. EXP refers to the animals cultured under a lower handling protocol. Asterisks show statistically significant difference between groups

The reduction of handling induced a significant (*p* = 0.0325) decrease of plasma cortisol concentration in Trial 1 (Fig. 4). The enzyme immunoassay experiment reported a mean concentration of 5.181 ± 1.413 ng cortisol/mL in the EXP group whereas the standard cultured CTRL group registered a mean value ten orders of magnitude higher with 54.32 ± 17.75 ng cortisol/mL.

The impact of the reducing handling protocol showed similar results in the pre-fattening stage in Trial 2. In this sub-experiment, the less disturbed animals from EXP group showed a mean of 4.140 ± 0.7075 ng cortisol/mL, lower to the values registered in the CTRL counterparts 10.58 ± 1.803 ng cortisol/mL. Statistical analysis revealed significant differences (*p* = 0.0088) between groups.

### Expression of stress-related and apoptosis genes

**Fig. 5:**
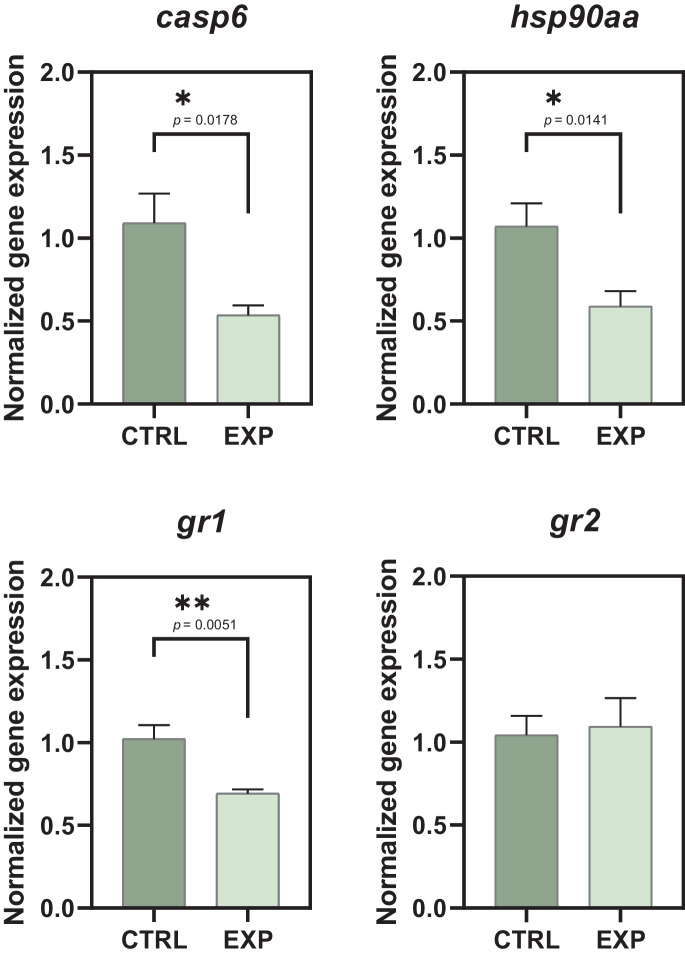
Relative expression of *casp6*, *hsp90aa*, *gr1*, and *gr2* in musculoskeletal samples from Trial 2 specimens (*n* = 8). CTRL refers to the animals standard cultured. EXP refers to the animals cultured under a lower handling protocol. Asterisks show statistically significant difference between groups

In the muscle samples from Trial 2 individuals, the relative gene expression of the glucocorticoid receptors 1 and 2 (*gr1* and *gr2*) showed different tendency (Fig. 5). On the one hand, qPCR analysis revealed a clear statistically significant *gr1* downregulation (*p* = 0.0051) in the EXP group. On the other hand, *gr2* did not show differences on expression levels (*p* > 0.0500) between the two culture protocols.

The expression of the gene encoding the chaperone Hsp90aa was also downregulated (*p* = 0.0141) in the less disturbed EXP group in Trial 2 after 4 months of culture. Following the same pattern, the analysis of the Caspase 6 gene, *casp6,* evidenced a significantly decrease (*p* = 0.0178) in its gene expression in the EXP samples compared to the CTRL (Fig. 5).

## Discussion

Both in their natural environment or in aquaculture facilities, fish deal with stress through the activation of the hypothalamic-pituitary-interrenal endocrine axis and, as an immediately consequence, corticosteroids are released into the blood. Cortisol is the predominant corticosteroid hormone in teleost fish and is an essential component of the stress response in this infraclass (Mommsen et al. 1999). It is known since decades ago that plasma cortisol levels rise during acute and chronic stress in fish species with commercial value (Pickering and Pottinger 1989; Wendelaar Bonga 1997; Van Weerd and Komen 1998) and nowadays it is vastly used as a molecular biomarker of stress response in aquaculture-focused experiments (Hanke et al. 2020; McLean 2021; Murugananthkumar and Sudhakumari 2022). Overall, our results showed that our proposed experimental protocol reducing husbandry events involving handling reduces plasma cortisol levels in both performed trials with specimens from the pre-fattening and the fattening culture stage (Fig. 4). Thus, the cortisol values shown here are in line with ranges previously recorded for this species for the two animal cohorts, both in the fattening stage of Trial 1 (Figueiredo et al. 2020) and in the pre-fattening stage of Trial 2 (Benítez-Dorta et al. 2017). The present study found evidence about the beneficial effect of a reduction of anthropogenic stimuli during handling in the levels of cortisol in Senegalese sole potentially promoting animal welfare since a lower stressful condition is correlated to lower plasma cortisol levels in this species either high density stocking (Wunderink et al. 2011), thermal stress (Benítez-Dorta et al. 2017), feeding regimen (Conde-Sieira et al. 2018; Herrera et al. 2019), rearing conditions (Figueiredo et al. 2019), environmental osmolarity changes (Arjona et al. 2009), or transport (Martín et al. 2017) among others. Interestingly, unlike the data collected in Trial 1 (Fig. 2), only in Trial 2, corresponding to the animals in the pre-fattening stage, a significant difference was found in all the biometric studied parameters (Fig. 3). We registered higher SGR and mean weight and length values in the tanks subjected to lower human handling. It should be noted that the trend in Trial 1, although not significant, points to greater growth in the experimental group in SGR values. In this trial only the mean body weight in the tanks revealed increased values in the experimental group. Feed conversion ratios were lower in both trials, further supporting the benefits of a lower handling protocol in this species (Supplementary material 2). Since glucocorticoids promote the mobilization via the de novo synthesis of high energy substrates (i.e., gluconeogenesis) and redistribution of energy in order to mitigate stress, the effects of chronic stress on growth in fish are generally been attributed to the actions of cortisol (Mommsen et al. 1999; Aluru and Vijayan 2009; Schreck and Tort 2016) and our results support this basis.

In order to further study the potential beneficial effects of the proposed protocol and to explore its impact on the specimens we performed gene expression analysis in musculoskeletal samples of fish from Trial 2, which reported a growth promotion. The gene expression of the Caspase 6 (*casp6*) showed a lower expression in the experimental animals (Fig. 5). Casp6 is a member of the evolutionarily conserved proteases family of the Caspases which play a key role in controlling apoptosis and inflammatory responses (McIlwain et al. 2013; Shalini et al. 2015). Numerous different caspases with specific functions have been described in teleost fish (Takle et al. 2006; Zeng et al. 2021). Based on physiological functions, Casp6 belongs to the apoptosis executioner caspases subgroup known as the principal mediators of apoptosis in all tissues (Spead et al. 2018) which act downstream of the apoptotic initiators. The overexpression of *casp6* gene under stressful conditions have been reported in different experimental approaches in fish with commercial interest. For example, in *Oncorhynchus mykiss* specimens exposed to confinement stress, *casp6* mRNA levels were reported higher than controls in head kidney samples (Laing et al. 2001) or peripheral blood leucocytes (Yada et al. 2019). Similarly, *Salmo salar* embryos exposed to hyperthermia as a source of stress, revealed *casp6* overexpression (Takle et al. 2006). In experiments involving *Solea senegalensis*, the exposure to the drug oxytetracycline, a significant increase in hepatic *casp6* gene transcription in juveniles was reported (Tapia-Paniagua et al. 2015) whereas the ingestion of the oestrogenic biological active isoflavone genistein in early life stages induced some temporal disrupting effects before metamorphosis (Sarasquete et al. 2018). Here, we have shown a reduction in the expression of this gene in the musculoskeletal tissue of the specimens grown under potentially lower stressful conditions which reported a better growth performance in the pre-fattening stage compared to the standard culture specimens. Along with the activation of caspases and apoptosis, the cell response to stressful biotic or abiotic stimuli includes numerous heat shock proteins (Hsp) interacting with denatured proteins (Srivastava 2002). The Hsp family consists on a highly conserved ubiquitous protein group typically expressed in response to environmental stress factors. In finfish and crustaceans, stressors such as toxins presence, pollutants, microbial-derived damage, protein degradation, anoxia, hypoxia, or acidosis can lead to their up-regulation (Roberts et al. 2010). Therefore, their overexpression has been broadly considered as stress indicator since decades ago. Here, we found a reduction on the *hsp90a* gene expression in the musculoskeletal samples from fish in the pre-fattening stage cultured in our experimental less-stressful protocol when compared to the routine culture (Fig. 5). Hsp90 proteins have been studied in depth in Senegalese sole and their main features and sequence identities with other organisms have been previously described (Manchado et al. 2008). In this flatfish, overexpression of *hsp90aa* has been reported after a thermal shock in post-metamorphic individuals (20 dah) (Manchado et al. 2008) and juveniles (Benítez-Dorta et al. 2017). As discussed with *casp6* results, *hp90a* downregulation data support our starting hypothesis since the proposed protocol positively modulates a molecular biomarker that ultimately positively effects on animal welfare. Moreover, Hsp90aa is a molecular chaperone that together with other cochaperones binds specific proteins creating complexes that helps target proteins to achieve or to maintain their active states (Grad and Picard 2007). One of the molecular target clients of Hsp90 are the glucocorticoid receptors (Grs) and it acts on regulating their correct maturation, trafficking and transcriptional regulation (Grad and Picard 2007). The glucocorticoid receptors, are ligand-regulated transcription factors that belong to the superfamily of nuclear receptors, bind glucocorticoids and regulates transcription of target genes via promoters, or enhancer binding (Mangelsdorf et al. 1995). In teleost, two different isoforms of the Gr are generally described for almost all species and together with the mineralocorticoid receptor (Mr) recognize cortisol (Bury and Sturm 2007; Faught et al. 2016). Both glucocorticoid receptors 1 and 2 (Gr1 and Gr2) expression has been previously described in *Solea senegalensis* muscle tissue (Benítez-Dorta et al. 2013). Moreover, this tissue has showed a clear response in terms of glucocorticoid receptor 1 gene expression overexpression under stress conditions such as repetitive chasing stress (Benítez-Dorta et al. 2013). In line with these published results, in our experiment we have found a clear lower expression of glucocorticoid receptor 1 in the muscle samples from fish cultured in a reduced handling protocol comparing to control (Fig. 5) indicating a reduction of stress in these animals at a molecular level. This modulation of *gr1* expression could be reasonably linked to the lower basal cortisol concentration values recorded in this experimental group. In other words, with a lower presence of the ligand in the blood, we registered a lower expression of the receptor in muscle target cells. The sum of these experimental evidences from the molecular point of view together with the improvement of the growth performance are of potential interest for the flatfish aquaculture producers since they allow laying the foundations to continue exploring protocols based on the reduction of handling and human interaction with the specimens in the culture facilities in order to impact on production optimization as well as animal welfare promotion.

## Conclusions

In this study, an experimental husbandry protocol involving lower handling events promoted the reduction of the cortisol levels in *Solea senegalensis* specimens in two production stages: pre-fattening and fattening. The reduction of this physiological biomarker of stress was also accompanied by a growth performance (SGR, mean body weight and mean length) of Senegalese sole individuals in the pre-fattening stage after 4 months of culture. These fish revealed also a reduction in the expression of key genes associated with the glucocorticoid signaling pathway and apoptosis (*gr1*, *hsp90*, and *casp6*) in musculoskeletal samples. Taken together, our results provide evidence that protocols focusing on decreasing the number of anthropogenic stimuli can be useful to promote animal welfare in production facilities and ultimately positively impact on the *Solea senegalensis* growth in the pre-fattening stage.

## Supplementary information

ESM 1 (DOCX 205 kb)

## Data Availability

Not applicable
