# Supplementary material for: Impact of different levels of handling on Solea senegalensis culture: effects on growth and molecular markers of stress
Source: Fish Physiol Biochem. 2023 Sep 21;50(5):1987–2000. doi: 10.1007/s10695-023-01239-9 (PMC11576817; doi:10.1007/s10695-023-01239-9)
Supplement: Supplementary file 1 — (DOCX 205 kb) [file 10695_2023_1239_MOESM1_ESM.docx]

**Supplementary material**

**Impact of different levels of handling on *Solea senegalensis* culture: effects on growth and molecular markers of stress.**

David G. Valcarce^1,2^, Marta F. Riesco^1^, Juan Manuel Martínez-Vázquez^2^, José Luis Rodríguez Villanueva^3^, Vanesa Robles^1*^

**Supplementary material 1.** Mean weight and mean length comparison (data from all the individuals included in the experimental groups) in Trial 1 (fattening stage) and Trial 2 (prefattening stage). CTRL refers to the animals standard cultured. EXP refers to the animals cultured under a lower handling protocol. Asterisks show statistically significant difference between groups.

**
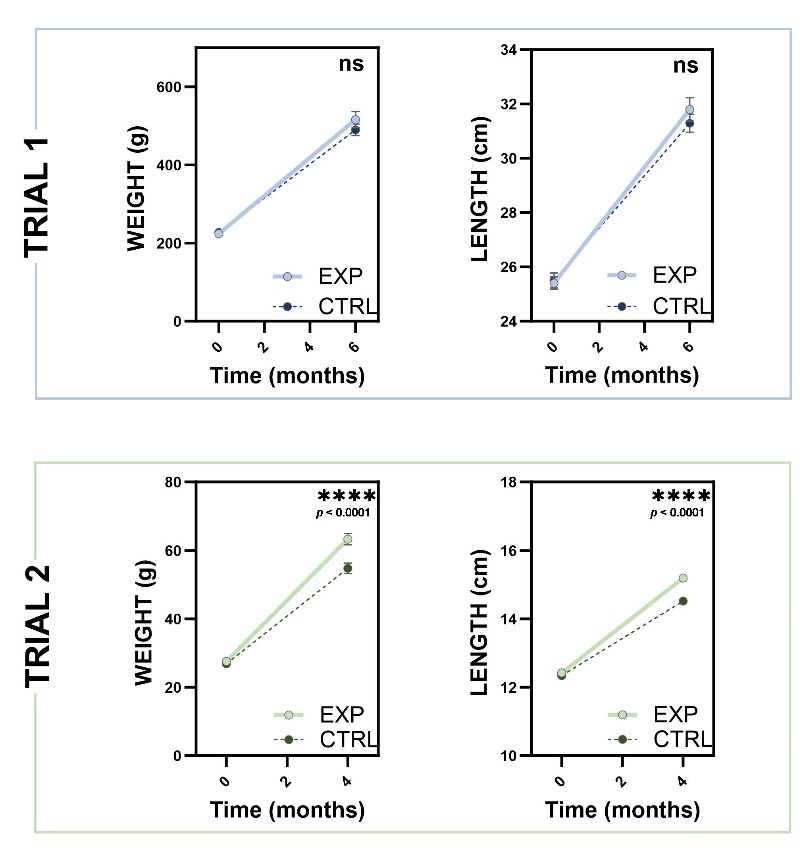
**

**Supplementary material 2.** Feed conversion ratios (FCR) for each Trial in the experiment: Trial 1 corresponds to specimens in the fattening culture stage and Trial 2 corresponds to specimens in the pre-fattening stage. FCR was calculated as the ratio of the total amount of feed (g) provided to the tanks during the experiment to the increment in the biomass (g) in the tank (final biomass-initial biomass) in each tank. CTRL refers to the animals standard cultured. EXP refers to the animals cultured under a lower handling human-animal interaction protocol. Asterisks show statistically significant difference between groups.

**
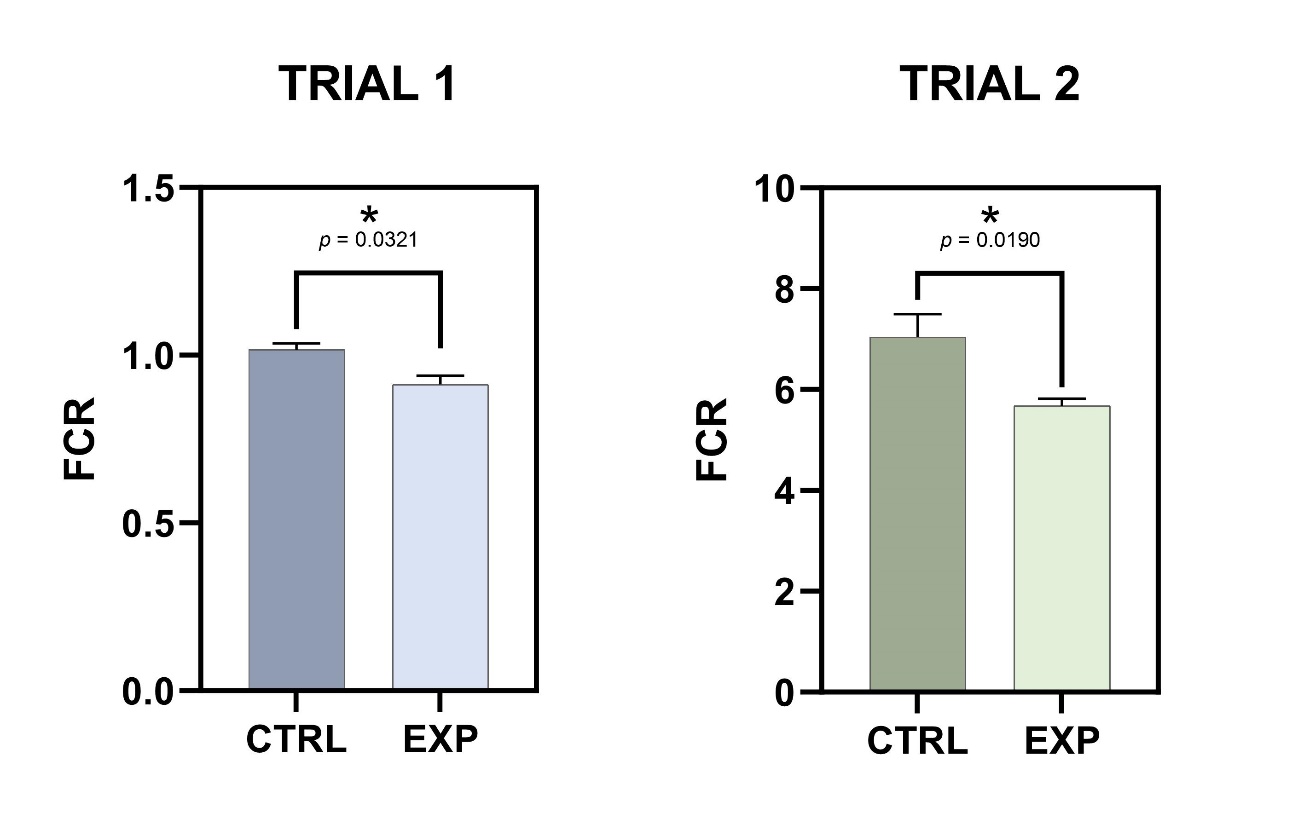
**
